# What you hear and see specifies the perception of a limb-respiratory-vocal act

**DOI:** 10.1098/rspb.2022.1026

**Published:** 2022-07-27

**Authors:** Wim Pouw, James A. Dixon

**Affiliations:** ^1^ Donders Institute for Brain Cognition and Behaviour, Radboud University, Nijmegen, Gelderland, The Netherlands; ^2^ Max Planck Institute for Psycholinguistics, Nijmegen, Gelderland, The Netherlands; ^3^ Department of Psychology, University of Connecticut, Storrs, CT, USA

A commentary on ‘Beat gestures influence which speech sounds you hear' [1].

Bosker & Peeters [1] report an extensive and well-executed demonstration of how perception of vocalic aspects of speech is a multimodal affair that can be instantiated by visual information about co-speech upper limb movement. This discovery of a ‘gesture McGurk effect' (see also [2]) can be considered as a fascinating extension of the classic McGurk effect which is originally obtained in relation to articulatory aspects of speech perception. Instead, the authors interpret their discovery as a logically distinct phenomenon.

Bosker and Peeters seem to hold that gesture and speech are causally *independent* modes of communication as the gesture McGurk effect does not reflect information originating from ‘the same communicative channel (i.e. articulation)' [1, p. 17] as is the case for the classic McGurk effect. The authors follow this through by accepting that the *dependence* of speech perception with gesture is achieved via ‘top-down' cognitive inference: ‘As such, what we perceive is the model of reality that our brains provide us by binding visual and auditory communicative input, and not reality itself' [1, p. 7].

To address the first issue, there is evidence that respiratory-vocalic aspects of speech such as the fundamental frequency and intensity are directly modulated by physical impulses that are produced by beat-like upper limb movements [3–6]. This modulation is attributed to upper limb movements recruiting a wider ensemble of posture-maintaining muscles around the trunk which are implicated with control of expiration [7–9] and thus vocalic aspects of speech [5,10]. What this means is that there is a causally dependent biophysical relationship between vocalic aspects of speech and upper limb movements. For the present gesture McGurk effect this means that beat gestures and vocalic aspects of speech have a causal connection in reality. Specifically, markers of lexical stress that are affected by rate of expiratory flow, such as intensity and F0, will be affected because of gesture-induced changes in sub-glottal pressure (for a detailed account see [11]). Given the that the rate of expiratory flow is less directly related to duration we also think it is less obviously connected to the biomechanical impulses that gestures generate.

The experimental manipulation in [1] is such that the real sound source and the gesture source are detached so as to be manipulated. Yet, this does not mean that perception of vocalic aspects of speech as affected by seeing gesture is therefore detached from reality; the perception is rooted in the real connection of gesture and speech. Echoing classic critiques of cognitivist interpretations of the classic McGurk effect, listeners are not attending to the sound alone they are perceiving a limb-vocalic speech act, and varying information about physical impulses of gesture interacts with audition in the perception of a more global array of multimodal information [12,13].

To conclude, this commentary is meant to guard against an overly cognitively complex interpretation of the relation between gesture and speech, both in perception and production. We have argued that this discovery of the gesture McGurk effect beautifully reflects that vocalic actions tend to be perceived by attuning to ‘simultaneous changes in the structure of multiple forms of ambient energy' [12 p. 196] much like the articulatory McGurk effect.

## Data Availability

This article has no additional data.
